# Supplementary material for: Development of a digital imaging analysis system to evaluate the treatment response in superficial infantile hemangiomas
Source: PLoS One. 2023 Mar 20;18(3):e0282274. doi: 10.1371/journal.pone.0282274 (PMC10027214; doi:10.1371/journal.pone.0282274)
Supplement: Attachment — Submitted filename: Comments.docx [file pone.0282274.s002.docx]

Dear Editor, I suggest the following changes:

- The manuscript needs to be refined by a native speaker.

**Introduction**

1. **Line 41.** references are not listed in order of appearance in the text
2. **Line 42.** It is not appropriate to begin a sentence with an abbreviation. It must be corrected.
3. **Line 66-96.** The source from where you received this sentence should be cited.
4. The introduction part is typically very lengthy and needs to be brief**.**

**Method**

1. The method section has been well written and organized
2. **Image acquisition:**

**Line 95.** Please do not use abbreviation in the method section or at least write the complete word once before using abbreviation

3. **Data acquisition:** It is best to write inclusion criteria and exclusion criteria under different subheadings.
4. **Ethical consideration:** Ethics approval Please provide the ethics approval no.

**Reference**

**The DOI should include references 4, 18, 20, 21, and 22.**

**Figure 2.** A, B, C and D must be placed to the top left of each section of the figure, NOT overlapping the image
