# Supplementary material for: Development of a digital imaging analysis system to evaluate the treatment response in superficial infantile hemangiomas
Source: PLoS One. 2023 Mar 20;18(3):e0282274. doi: 10.1371/journal.pone.0282274 (PMC10027214; doi:10.1371/journal.pone.0282274)
Supplement: Attachment — Submitted filename: Response to Reviewer Comments.docx [file pone.0282274.s003.docx]

Dear Editor,

Thank you for reviewing our manuscript entitled "*Digital imaging analysis system evaluating treatment response in superficial infantile hemangiomas*". We appreciate your constructive comments and suggestions and have considered them in revising our work. Here is a point-by-point response to the reviewers' comments and concerns.

# Comment 1:

**The manuscript needs to be refined by a native speaker.**

*We appreciate your suggestion that the manuscript needs to be refined by a native speaker. We have asked a native English speaker to help us polish our article. We believe that this revision has greatly improved the readability and clarity of our manuscript.*

# Comment 2: I**ntroduction**:

A. **Line 41. references are not listed in order of appearance in the text.**

*We agree with the reviewer's assessment and have revised the manuscript to ensure that all references are listed in the order of appearance within the text.*

B. **Line 42. It is not appropriate to begin a sentence with an abbreviation. It must be corrected.**

*We appreciate your attention to detail and your suggestion regarding the use of abbreviations at the beginning of sentences. We have reviewed the text and made the necessary corrections to ensure that abbreviations are only used after they have been fully spelled out and defined.*

C. **Line 66-96. The source from where you received this sentence should be cited.**

*As suggested by the reviewer, we have added more references to support the statements in lines 66- 96. (references 8 and 18).*

D. **The introduction part is typically very lengthy and needs to be brief.**

***We have carefully reviewed the section and removed any redundant or unnecessary information while still maintaining the essential elements required for a comprehensive understanding of the background and context of the study.***

# **Comment 3: Method**

A. **The method section has been well-written and organized. Image acquisition. Line 95. Please do not use abbreviations in the method section, or at least write the complete word once before using abbreviations**

*We agree with the reviewer's assessment. We have reviewed the text to ensure that all abbreviations are defined at first occurrence within the text.*

B. **Data acquisition: It is best to write inclusion criteria and exclusion criteria under different subheadings.**

*We think this is an excellent suggestion and have now included the inclusion and exclusion criteria under a new subheading.*

C. **Ethical consideration: Ethics approval Please provide the ethics approval no.**

*As suggested by the reviewer, we have provided the ethics number in the manuscript.*

# **Comment 4: References**

****The DOI should include references 4, 18, 20, 21, and 22.****

***Reference 4 was obtained from a book; therefore, the doi is unavailable.***

***References 19 (previous 18), 21 (previous 20), and 22 (previous 21) are patents. We have now amended the format of these references to align with the PLOS ONE guidelines.***

***Reference 23 (previous 22) was obtained from a journal. We have now included the DOI for this reference.***

# ****Comment 5: Figures****

**Figures 2. A, B, C, and D must be placed to the top left of each section of the figure, NOT overlapping the image**

*We have now reformatted figure 2 so that the labels do not obscure the images.*

Once again, we would like to thank you for your time and expertise, and we hope that the revised manuscript meets your expectations.

Sincerely,

Qian Liu

The First Affiliated Hospital of GanNan Medical University

Jiangxi University of Traditional Chinese Medicine

Ganzhou, Jiangxi, China

liuqianjxzyy@163.com
